# The Albedo of Ryugu: Evidence for a High Organic Abundance, as Inferred from the Hayabusa2 Touchdown Maneuver

**DOI:** 10.1089/ast.2019.2198

**Published:** 2020-07-08

**Authors:** Christian Potiszil, Ryoji Tanaka, Katsura Kobayashi, Tak Kunihiro, Eizo Nakamura

**Affiliations:** The Pheasant Memorial Laboratory for Geochemistry and Cosmochemistry, Institute for Planetary Materials, Okayama University, Misasa, Tottori, Japan.

**Keywords:** Hayabusa2, Ryugu, Sample return, Organic matter, Albedo. Astrobiology 20, 916–921

## Abstract

The Hayabusa2 mission successfully collected samples from the asteroid Ryugu last year and will return these to Earth in December 2020. It is anticipated that the samples will enable the analysis of terrestrially uncontaminated organic matter and minerals. Such analyses are in turn expected to elucidate the evolution of organic matter through Solar System history, including the origination and processing of biogenically important molecules, which could have been utilized by the first organisms on Earth. In anticipation, studies have made predictions concerning the properties of Ryugu, including its composition. The spectral characteristics of Ryugu, such as albedo, have been employed to relate the asteroid to members of the carbonaceous chondrite group that have been identified on Earth. However, the recent Hayabusa2 touchdown highlights a disparity between the color of surfaces of displaced platy fragments, indicating a brightening trend for the surface exposed to space compared to that facing into the body. Here we present a mass balance calculation with reference to data from the literature, which indicates that Ryugu may contain a significantly higher abundance of organic matter (likely >50%) than the currently most accepted meteorite analogues. A high organic content may result in high levels of extractable organic matter for the second touchdown site, where the spacecraft sampled freshly exposed material. However, high abundances of insoluble aromatic/graphitic rich organic matter may be present in the first touchdown site, which sampled the surface of Ryugu that had been exposed to space. Moreover, we suggest that the potentially high organic abundance and the rubble-pile nature of Ryugu may originate from the capture of rocky debris by a comet nucleus and subsequent water-organic-mineral interactions and sublimation of water ice.

## 1. Introduction

Ryugu has been classified as a C-type asteroid (Tsuda *et al.,* 2013) and is the main target of the Hayabusa2 mission. Due to the classification of Ryugu, it is expected that the returned samples will contain primitive organic matter (Watanabe *et al.,* 2017). Organic compounds related to life on Earth have been found previously in carbonaceous chondrites, such as Murchison (CM2) (Sephton, 2002, 2013). Unfortunately, terrestrial contamination represents a persistent problem to the interpretation of extraterrestrial organic matter from meteorites. It is hoped that the Hayabusa2 mission will provide a contaminant-free sample for study in laboratories on Earth.

In anticipation of the Ryugu samples, which will be returned in December 2020, studies have attempted to understand the asteroid's composition. Ryugu has been recently classified as a Cb-type asteroid (Sugita *et al.,* 2019); such bodies are thought to be related to unheated comet-like icy asteroids (Vernazza *et al.,* 2015). Such asteroids have been further linked to interplanetary dust particles (IDPs), which are rich in organic matter (up to 90% by volume) (Flynn *et al.,* 2004). Nevertheless, the exceptionally low albedo of Ryugu (0.20–1.88%) has been explained through a similarity to dehydrated or heated carbonaceous chondrites (Perna *et al.,* 2017; Sugita *et al.,* 2019) or the CM2 chondrite Mighei (Le Corre *et al.,* 2018). If Ryugu was of a heated CM2 composition, it would be expected to contain almost no organic matter, depending on the degree of heating, due to the conversion of organic carbon to graphite. Alternatively, in the case of a Mighei-like composition, Ryugu would be expected to contain ∼2.60–2.85 wt % organic carbon (Kerridge, 1985).

Footage of the surface of Ryugu was obtained from a recent touchdown maneuver (JAXA, July 11, 2019). The video captured by the camera shows that disturbed platy material is significantly darker on one side and a similar color to the surface of Ryugu on the other, indicating that Ryugu is darker beneath its surface. The disparity of color is clearest within objects that are spinning as a result of the Hayabusa2 touchdown. Studies have demonstrated that while silicates such as olivine and pyroxene get darker on exposure to irradiation, such as that from the solar wind (space weathering), organic matter gets brighter at the visible light band of 0.55 μm (Moroz *et al.,* 2004; Lantz *et al.,* 2017). The darkening of silicates is due to the formation of Fe nanoparticles (Lantz *et al.,* 2017), whereas the brightening of organic matter is thought to result from its partial conversion to graphite (Moroz *et al.,* 2004). Therefore, the observed brightening of Ryugu's exposed surface, compared to its interior, may arise from space weathering of organic matter. Currently, all the suggested analogues for Ryugu employ compositions that are relatively organic poor, in comparison to IDPs for example. Instead, the observation highlighted above indicates that Ryugu may contain a greater abundance of organic matter than currently hypothesized.

Here, we present a mass balance calculation, using data from the literature, to predict the potential organic/mineral composition of Ryugu. This information can help prepare terrestrial laboratories for the samples of Ryugu, in terms of what analyses and procedures might be applicable and necessary. Furthermore, we outline a potential model for the origin of Ryugu and its organic and inorganic components.

## 2. Calculating the Surface Composition of Ryugu

Currently it is expected that Ryugu will contain organic matter within the range of carbonaceous chondrites (likely <10 wt %) (Kerridge, 1985) or even less if it has experienced sufficient heating. However, as outlined above, the current proposed analogues for Ryugu do not entirely match the observed characteristics of this asteroid. One possible explanation, for the color disparity of displaced fragments and the low albedo, could be a higher organic matter abundance in Ryugu than in carbonaceous chondrites. Accordingly, the current study will try to determine what organic contents could explain the albedo of Ryugu through a series of mass balances.

Firstly, appropriate mineral and organic components must be selected. Such components should be representative of the surface conditions of Ryugu. Previous studies have irradiated both carbonaceous chondrites and organic matter to simulate space weathering (Moroz *et al.,* 2004; Lantz *et al.,* 2017). Although these studies utilize different energies and fluencies to irradiate their materials than the composition of the solar wind, such studies inform us about the probable effects of long periods of exposure.

The organic matter chosen by Moroz *et al.* (2004), for their space weathering studies, included asphaltite, which is composed of polyaromatic cores with aliphatic, phenolic, and carboxyl groups. Therefore, it contains within its structure similar moieties to the macromolecular organic matter (MOM) that is found in carbonaceous chondrites (Sephton, 2013). Other experimental studies have attempted to generate organic matter similar to the optical properties of distant Solar System bodies through irradiation of either gaseous atmospheres or surface ice deposits (Cruikshank *et al.,* 2005). The organic products of these experiments are tholins, which have been shown to represent the low albedo of Kuiper Belt objects and areas of Pluto (Cruikshank *et al.,* 2005, 2015). Therefore, both tholins and asphaltites may be representative of the types of organics found at the surface of Ryugu.

Accordingly, appropriate parameters for the mass balance calculations have been taken from the literature. Sugita *et al.* (2019) measured the geometric albedo for Ryugu at 0.55 μm and determined this to be 4.5 ± 0.2%. To compare this value to ground-based measurements of meteorites, the value was converted to a disk-integrated surface reflectance (1.88 ± 0.17%). Perna *et al.* (2017) also reported surface reflectance data for Ryugu from the X-shooter instrument, which was similarly corrected for comparison with spectra acquired in laboratories. In our calculations, values were required for the albedo of Ryugu that were comparable with lab-based studies of meteorites. Accordingly, we used the visible light spectrum reflectance values at 0.55 μm (0.20 ± 0.01% or 1.88 ± 0.17%) (Perna *et al.,* 2017; Sugita *et al.,* 2019) due to the relevance of visible light to the Hayabusa2 touchdown video. Albedo values, at 0.55 μm, were selected for irradiated meteorites (4.31 ± 0.14% for Mighei and 2.14 ± 0.11% for Tagish lake) (Lantz *et al.,* 2017) and organic matter (0.043% for irradiated asphaltite, 0.03% for BB [black to blue color] tholin, and 0.13% for YO [yellow to orange color] tholin) (Moroz *et al.,* 2004; Bernard *et al.,* 2006). The tholin data used in the current study relates to synthetic residues taken from near the gas inlet point (BB) and in the center of the reaction vessel (YO) (Bernard *et al.,* 2006). Note that the irradiated meteorites were used as analogues for the mineral component of Ryugu, and the standard deviation for error propagation was taken from Fig. 10 in the work of Lantz *et al.* (2017). The errors for the albedo of organic matter were not given in the literature, so the same percentage error was applied to these materials as for the meteorites. The calculated standard deviations are as follows: ±0.002, ±0.001, and ±0.005 for the albedo of irradiated asphaltite, BB, and YO tholins, respectively.

To calculate the theoretical organic compositions of Ryugu, we first defined a mass balance calculation to obtain the albedo of the mineral component of the irradiated meteorites, using the weight fraction of organic carbon for Mighei and Tagish Lake (2.73 ± 0.18 wt % for Mighei and 2.62 ± 0.18 wt % for Tagish Lake) (Kerridge, 1985; Grady *et al.,* 2002) (Eq. 1.). Note that the error was obtained from the two values given in the literature for each meteorite.

**Figure:**
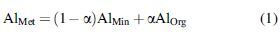


where α represents the fraction of the organic component of the meteorite (Kerridge, 1985; Grady *et al.,* 2002) and Al_Met_, Al_Min_, and Al_Org_ the albedo in reflectance (%) of the meteorite of interest, mineral and organic component at 0.55 μm, respectively.

Subsequently, we defined a mass balance calculation for obtaining the albedo of Ryugu (Eq. 2).

**Figure:**
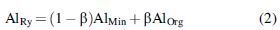


where β represents the fraction of the organic component of Ryugu and Al_Ry_ the albedo in reflectance (%) of Ryugu at 0.55 μm.

Both Eq. 1 and 2 were then combined to give a simplified equation (Eq. 3), which solves for the organic content of Ryugu (β).

**Figure:**
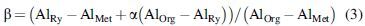


The calculated organic abundances for the various scenarios are given in Table 1. Errors for the data in Table 1 were calculated by propagating the errors according to Eq. 3.

**Table 1.** Calculated Organic Contents for Ryugu Based upon Different Organic (Asphaltite, Moroz *et al.,* 2004; BB and YO Tholin, Bernard *et al.,* 2006) and Meteorite (Lantz *et al.,* 2017) Compositions

Meteorite component	Organic component	Perna		Sugita	
		Value	SD	Value	SD
Mighei	Asphaltite	96.4	0.2	58.1	4.1
	BB Tholin	96.1	0.2	58.0	4.1
	YO Tholin	98.4	0.2	59.3	4.2
Tagish Lake	Asphaltite	92.9	0.5	14.8	9.1
	BB Tholin	92.1	0.6	14.6	9.0
	YO Tholin	96.8	0.5	15.3	9.5

Note that two values are given, one using the albedo of Ryugu taken from the work of Perna *et al.* (2017) (Perna) and the other from the work of Sugita *et al.* (2019) (Sugita). SD = standard deviation.

## 3. Discussion

### 3.1. Organic content

Although the calculated organic components differ significantly depending on the albedo assigned to Ryugu and the mineral component chosen, the values are still higher than those found in carbonaceous chondrites (Kerridge, 1985; Grady *et al.,* 2002). It is possible that the irradiation conditions used by the simulation studies do not entirely reflect the exposure of Ryugu to the solar wind, due to the higher energies that were employed, the unknown exposure age of Ryugu, and the lack of heavier ions. Nevertheless, such simulations should provide a rough indication of how Solar System materials would change with respect to irradiation from exposure to the solar environment. The results for the tholins, unsurprisingly as a result of their similar albedos, represent a distinct similarity to the values calculated for irradiated asphaltites, which is likely due to their synthesis involving irradiation.

The values calculated in Table 1 cover a large range of organic compositions (14.6–98.4%). The main parameters that dictate the difference in the values are the albedo of Ryugu at 0.55 μm and the irradiated meteorite albedo. In terms of the albedo of Ryugu, it may be safer to assume that the value of Sugita *et al.* (2019) is more reliable than that of Perna *et al.* (2017), due to the data being obtained from instruments on board Hayabusa2 rather than from a ground-based telescope. If the aforementioned is correct, a range of between ∼15 and 59% organic matter for Ryugu would be most appropriate. Moreover, the range could be further constrained by considering which meteorite component would be most applicable to Ryugu. Previous studies (Perna *et al.,* 2017; Le Corre *et al.,* 2018) have indicated that the silicate portion of Ryugu is most similar to CM2 chondrites, and as such Mighei is likely more appropriate in terms of its mineral component. Therefore, a range of ∼58–59% organic matter would seem most appropriate for the surface of Ryugu.

**FIG. 1.**
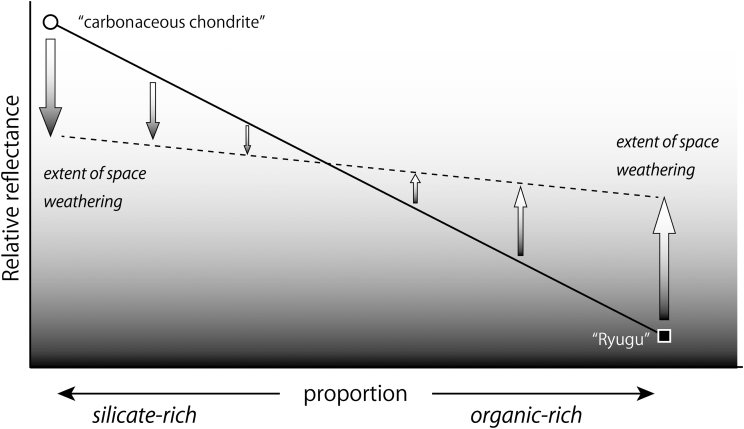
A cartoon illustrating the different effects of space weathering on silica-rich materials (carbonaceous chondrite-like) and organic-rich materials. While organic-rich materials are darker than silicate-rich materials, they become brighter upon irradiation, whereas silicate-rich materials, such as carbonaceous chondrites, become darker.

The results of the mass balance calculations indicate that the organic content within the surface of Ryugu may in fact be considerably higher than that of known carbonaceous chondrites (up to ∼10 wt %)(Kerridge, 1985). The broad range of calculated organic compositions falls within that of IDPs (Flynn *et al.,* 2004) and suggests that the similarity between their albedo and that of Ryugu (Sugita *et al.,* 2019) could relate to the presence of high abundances of organic matter. If the aforementioned is true, then Ryugu cannot simply be of a heated carbonaceous chondrite composition, because such meteorites do not contain sufficient organic matter. Indeed, an organic-rich Ryugu would be expected to become brighter on exposure to solar irradiation (Fig. 1), which could explain the disparity in color between different sides of material displaced by Hayabusa2 (JAXA, 2019).

### 3.2. Implications for terrestrial analyses

A high organic content will have considerable implications for the kinds of analyses that can be undertaken by terrestrial laboratories. Some more inorganic-focused techniques, such as laser ablation–inductively coupled plasma–mass spectrometry (LA-ICP-MS), scanning electron microscopy (SEM), and electron microprobe analysis (EPMA) may be limited by the small relative abundance of silicate minerals. However, other spatial analysis techniques, including Fourier transform infrared (FTIR) and Raman spectroscopy, secondary ion mass spectrometry (SIMS), transmission electron microscopy (TEM), and desorption electrospray ionization–orbitrap–mass spectrometry (DESI-OT-MS) (Potiszil *et al.,* 2020), may benefit from the increased organic content. A larger organic abundance may thus aid in one of the main objectives of the Hayabusa2 mission: to further our understanding of extraterrestrial organic matter.

The form of the organic matter in the Ryugu samples will greatly affect the types of analyses that can be applied. If Ryugu organic matter closely resembles the free/soluble and labile organic matter (FOM/SOM and LOM) fractions in carbonaceous chondrites, in terms of abundance, then a high organic content may mean compound-specific techniques can be applied successfully for very small sample sizes. Nevertheless, it may be the case that samples taken from the exposed regolith, during the first touchdown, contain high abundances of graphitized and MOM, due to the long-term effects of solar irradiation. Conversely, samples collected during the second touchdown may provide higher levels of extractable organic material, due to the removal of surface material by the Hayabusa2 impactor. Therefore, the sample locations may also be important when selecting the appropriate analytical techniques for the study of Hayabusa2 samples. Thus, future studies should attempt to understand the applicability of different techniques to samples of differing organic abundances and compositions.

An increased organic component in Ryugu samples may also mean they become more representative of the whole organic composition at smaller sample sizes (Carter and Sephton, 2013). Such a relationship between organic content and sample representativeness should be investigated to take into account the potential for higher organic contents. If smaller sample sizes can be used with confidence that they will reflect the returned sample composition overall, then more sample can be conserved for further analysis.

### 3.3. Implications for the formation of Ryugu

The results of this study indicate that Ryugu may contain between ∼15 and 98% organic matter depending on the parameters used for the calculations. If the silicate component is more similar to a CM2 composition, as has been suggested (Perna *et al.,* 2017; Le Corre *et al.,* 2018), then the organic composition calculated by the current study would be expected to be >50%. Furthermore, the large disparity between the light and dark sides of displaced platy material, from the Hayabusa2 touchdown, indicates a considerable component of the material has been altered by space weathering, a trend that can only be explained by the alteration of a large amount of organic matter. Such a high organic composition, similar to that of IDPs, has implications for the formation of Ryugu. In a scenario where organic matter is >50%, the formation of Ryugu cannot be solely explained by dehydration or heating of a CM or CI composition, as was suggested previously (Perna *et al.,* 2017; Sugita *et al.,* 2019). However, as has also been indicated (Sugita *et al.,* 2019), the presence of boulders on Ryugu rules out the entire body being simply of an IDP composition. Therefore, an alternative hypothesis for the origin of Ryugu would be beneficial to the discussion surrounding its formation.

Ryugu is a Cb-type asteroid, and such bodies likely contained high abundances of ice in their past. Indeed, Vernazza *et al.* (2015) described Cb-type asteroids as comet-like. The parent body of the Chelyabinsk meteorite has been indicated as a comet that captured debris resulting from a high-velocity collision, thus forming a rubble pile after water ice sublimation (Nakamura *et al.,* 2019). The presence of organic matter (Popova *et al.,* 2013) and evidence of aqueous alteration were found within the Chelyabinsk meteorite, which were indicated as the result of interactions between the cometary ice and organic matter and the captured silicate minerals (Nakamura *et al.,* 2019). If we apply the same scenario to Ryugu, it may help explain the presence of high organic matter abundances at the surface and the presence of CM-like fragments (the boulders), which could have become partially aqueously altered.

Comets have highly eccentric orbits, which means they periodically come very close to the Sun. During this period, it may be possible for solid ice to melt and give rise to organic-rich aqueous fluids close to the surface of the body. If Ryugu was indeed a rubble pile captured by a comet, then interactions between organic-rich ices and silicate minerals could yield more complex organic matter (Schulte and Shock, 2004). Accordingly, relationships between phyllosilicates and organic matter have been widely observed in carbonaceous chondrites (Pearson *et al.,* 2002; Le Guillou and Brearley, 2014; Yesiltas and Kebukawa, 2016; Potiszil *et al.,* 2020). Furthermore, it has been demonstrated that certain extraterrestrial organic compounds can form polymers on exposure to aqueous alteration conditions (Vinogradoff *et al.,* 2018).

Over many cycles, the organic composition of the comet-like Ryugu parent body could have evolved to give rise to much higher abundances of nonvolatile organic matter than originally present. With time the volatile ices could be lost from Ryugu through sublimation. During this period, vibrations imparted to the comet through expansion and contraction, as a result of being close to and far from the Sun, as well as impacts and interaction with other bodies, could move silicate material toward its center. With time, both the radius and mass of Ryugu would decrease, through sublimation, but the silicate fragments would retain the angular momentum in the core. Consequently, the body's rotational velocity would increase as a result of the conservation of angular momentum and thus give rise to its abacus bead shape. Sublimation could also concentrate the nonvolatile MOM at the surface of Ryugu and possibly within cracks and cavities in its interior, in a similar way to freeze-drying (*e.g.,* Hoang *et al.,* 2019). Therefore, the potentially high organic abundance, visible albedo, and rubble-pile nature of Ryugu may originate from interactions between rocky and icy solar bodies, in a similar way to the model proposed for the Chelyabinsk meteorite (Nakamura *et al.,* 2019). Such a scenario for the formation of Ryugu has an advantage over other suggestions, because it can explain both the IDP-like albedo and potentially high organic content of Ryugu, as well as the presence of partially hydrated material that could constitute the boulders observed on its surface and likely within its interior. Furthermore, the lack of large organic-rich meteorites, among Earth's collections, is likely due to their breakup and burning on entry into Earth's oxygen-rich atmosphere.

The hypothesis outlined here, that Ryugu may contain significantly higher organic abundances than currently indicated by the closest meteorite analogues, will be tested upon the delivery of Ryugu samples to Earth. The Pheasant Memorial Laboratory (PML) (Nakamura *et al.,* 2003) at the Institute for Planetary Materials, Okayama University at Misasa, previously received Hayabusa samples from the asteroid Itokawa. An initial comprehensive analysis of fine particles was successfully performed (Nakamura *et al.,* 2012). PML has been designated as the phase-2 curation facility for the Hayabusa2 mission. PML will receive Ryugu samples, and work is underway to prepare for their comprehensive analysis.

## Abbreviations Used

BB: black to blue color
IDPs: interplanetary dust particles
MOM: macromolecular organic matter
PML: Pheasant Memorial Laboratory
YO: yellow to orange color

## Notes

Associate Editor: Norman Sleep

## References

[B1] Bernard J., Quirico E., Brissaud O., Montagnac G., Reynard B., McMillan P., Coll P., Nguyen M., Raulin F., and Schmitt B. (2006) Reflectance spectra and chemical structure of Titan's tholins: application to the analysis of Cassini–Huygens observations. Icarus 185:301–307

[B2] Carter J.N. and Sephton M.A. (2013) A Bayesian statistical assessment of representative samples for asteroidal or meteoritical material. Meteorit Planet Sci 48:976–996

[B3] Cruikshank D.P., Imanaka H., and Dalle Ore C.M. (2005) Tholins as coloring agents on outer Solar System bodies. Adv Space Res 36:178–183

[B4] Cruikshank D.P., Grundy W.M., DeMeo F.E., Buie M.W., Binzel R.P., Jennings D.E., Olkin C.B., Parker J.W., Reuter D.C., Spencer J.R., Stern S.A., Young L.A., and Weaver H.A. (2015) The surface compositions of Pluto and Charon. Icarus 246:82–92

[B5] Flynn G., Keller L., Jacobsen C., and Wirick S. (2004) An assessment of the amount and types of organic matter contributed to the Earth by interplanetary dust. Adv Space Res 33:57–66

[B6] Grady M.M., Verchovsky A.B., Franchi I.A., Wright I.P., and Pillinger C.T. (2002) Light dement geochemistry of the Tagish Lake CI2 chondrite: comparison with CI1 and CM2 meteorites. Meteorit Planet Sci 37:713–735

[B7] Hoang Q.D., Kunihiro T., Sakaguchi C., Yamanaka M., Kitagawa H., and Nakamura E. (2019) Determination of abundances of fifty-two elements in natural waters by ICP-MS with freeze-drying pre-concentration. Geostand Geoanal Res 43:147–161

[B8] JAXA. (2019) Hayabusa2 1st Touchdown. Available online at https://www.youtube.com/watch?v=-3hO58HFa1M

[B9] Kerridge J.F. (1985) Carbon, hydrogen and nitrogen in carbonaceous chondrites: abundances and isotopic compositions in bulk samples. Geochim Cosmochim Acta 49:1707–1714 11539652 10.1016/0016-7037(85)90141-3

[B10] Lantz C., Brunetto R., Barucci M.A., Fornasier S., Baklouti D., Bourçois J., and Godard M. (2017) Ion irradiation of carbonaceous chondrites: a new view of space weathering on primitive asteroids. Icarus 285:43–57

[B11] Le Corre L., Sanchez J.A., Reddy V., Takir D., Cloutis E.A., Thirouin A., Becker K.J., Li J.-Y., Sugita S., and Tatsumi E. (2018) Ground-based characterization of Hayabusa2 mission target asteroid 162173 Ryugu: constraining mineralogical composition in preparation for spacecraft operations. Mon Not R Astron Soc 475:614–623

[B12] Le Guillou C. and Brearley A. (2014) Relationships between organics, water and early stages of aqueous alteration in the pristine CR3.0 chondrite MET 00426. Geochim Cosmochim Acta 131:344–367

[B13] Moroz L., Baratta G., Strazzulla G., Starukhina L., Dotto E., Barucci M.A., Arnold G., and Distefano E. (2004) Optical alteration of complex organics induced by ion irradiation: 1. Laboratory experiments suggest unusual space weathering trend. *Icarus* 170:214–228

[B14] Nakamura E., Makishima A., Moriguti T., Kobayashi K., Sakaguchi C., Yokoyama T., Tanaka R., Kuritani T., and Takei H. (2003) Comprehensive geochemical analyses of small amounts (< 100 mg) of extraterrestrial samples for the analytical competition related to the sample return mission MUSES-C. Inst Sp Astronaut Sci Rep SP 16:49–101

[B15] Nakamura E., Makishima A., Moriguti T., Kobayashi K., Tanaka R., Kunihiro T., Tsujimori T., Sakaguchi C., Kitagawa H., Ota T., Yachi Y., Yada T., Abe M., Fujimura A., Ueno M., Mukai T., Yoshikawa M., and Kawaguchi J. (2012) Space environment of an asteroid preserved on micrograins returned by the Hayabusa spacecraft. Proc Natl Acad Sci USA 109:E624–E629 22371561 10.1073/pnas.1116236109 PMC3306700

[B16] Nakamura E., Kunihiro T., Ota T., Sakaguchi C., Tanaka R., Kitagawa H., Kobayashi K., Yamanaka M., Shimaki Y., Bebout G.E., Miura H., Yamamoto T., Malkovets V., Grokhovsky V., Koroleva O., and Litasov K. (2019) Hypervelocity collision and water-rock interaction in space preserved in the Chelyabinsk ordinary chondrite. Proc Jpn Acad Ser B Phys Biol Sci 95:165–177 10.2183/pjab.95.013 PMC6541723 30971619

[B17] Pearson K., Sephton A., Kearsley T., Bland A., and Franchi A. (2002) Clay mineral–organic matter relationships in the early Solar System. Meteorit Planet Sci 37:1829–1833

[B18] Perna D., Barucci M.A., Ishiguro M., Alvarez-Candal A., Kuroda D., Yoshikawa M., Kim M.-J., Fornasier S., Hasegawa S., Roh D.-G., Müller T.G., and Kim Y. (2017) Spectral and rotational properties of near-Earth asteroid (162173) Ryugu, target of the Hayabusa2 sample return mission. Astron Astrophys 599, doi:10.1051/0004-6361/201630346

[B19] Popova O.P., Jenniskens P., Emel'yanenko V., Kartashova A., Biryukov E., Khaibrakhmanov S., Shuvalov V., Rybnov Y., Dudorov A., Grokhovsky V.I., Badyukov D.D., Yin Q.-Z., Gural P.S., Albers J., Granvik M., Evers L.G., Kuiper J., Kharlamov V., Solovyov A., Rusakov Y.S., Korotkiy S., Serdyuk I., Korochantsev A.V., Larionov M.Y., Glazachev D., Mayer A.E., Gisler G., Gladkovsky S.V., Wimpenny J., Sanborn M.E., Yamakawa A., Verosub K.L., Rowland D.J., Roeske S., Botto N.W., Friedrich J.M., Zolensky M.E., Le L., Ross D., Ziegler K., Nakamura T., Ahn I., Lee J.I., Zhou Q., Li X.-H., Li Q.-L., Liu Y., Tang G.-Q., Hiroi T., Sears D., Weinstein I.A., Vokhmintsev A.S., Ishchenko A.V., Schmitt-Kopplin P., Hertkorn N., Nagao K., Haba M.K., Komatsu M., and Mikouchi T. (2013) Chelyabinsk airburst, damage assessment, meteorite recovery, and characterization. Science 342:1069–1073 24200813 10.1126/science.1242642

[B20] Potiszil C., Tanaka R., Ota T., Kunihiro T., Kobayashi K., and Nakamura E. (2020) Concentration of meteoritic free organic matter by fluid transport and adsorption. Geochem Perspect Lett 13, doi:10.7185/geochemlet.2010

[B21] Schulte M. and Shock E. (2004) Coupled organic synthesis and mineral alteration on meteorite parent bodies. Meteorit Planet Sci 39:1577–1590

[B22] Sephton M.A. (2002) Organic compounds in carbonaceous meteorites. Nat Prod Rep 19:292–311 12137279 10.1039/b103775g

[B23] Sephton M.A. (2013) Organic geochemistry of meteorites. In *Treatise on Geochemistry: Second Edition,* edited by H.D. Holland and K.K. Turekian, Elsevier, Oxford, UK, pp 1–31

[B24] Sugita S., Honda R., Morota T., Kameda S., Sawada H., Tatsumi E., Yamada M., Honda C., Yokota Y., Kouyama T., Sakatani N., Ogawa K., Suzuki H., Okada T., Namiki N., Tanaka S., Iijima Y., Yoshioka K., Hayakawa M., Cho Y., Matsuoka M., Hirata N., Hirata N., Miyamoto H., Domingue D., Hirabayashi M., Nakamura T., Hiroi T., Michikami T., Michel P., Ballouz R.-L., Barnouin O.S., Ernst C.M., Schröder S.E., Kikuchi H., Hemmi R., Komatsu G., Fukuhara T., Taguchi M., Arai T., Senshu H., Demura H., Ogawa Y., Shimaki Y., Sekiguchi T., Müller T.G., Hagermann A., Mizuno T., Noda H., Matsumoto K., Yamada R., Ishihara Y., Ikeda H., Araki H., Yamamoto K., Abe S., Yoshida F., Higuchi A., Sasaki S., Oshigami S., Tsuruta S., Asari K., Tazawa S., Shizugami M., Kimura J., Otsubo T., Yabuta H., Hasegawa S., Ishiguro M., Tachibana S., Palmer E., Gaskell R., Le Corre L., Jaumann R., Otto K., Schmitz N., Abell P.A., Barucci M.A., Zolensky M.E., Vilas F., Thuillet F., Sugimoto C., Takaki N., Suzuki Y., Kamiyoshihara H., Okada M., Nagata K., Fujimoto M., Yoshikawa M., Yamamoto Y., Shirai K., Noguchi R., Ogawa N., Terui F., Kikuchi S., Yamaguchi T., Oki Y., Takao Y., Takeuchi H., Ono G., Mimasu Y., Yoshikawa K., Takahashi T., Takei Y., Fujii A., Hirose C., Nakazawa S., Hosoda S., Mori O., Shimada T., Soldini S., Iwata T., Abe M., Yano H., Tsukizaki R., Ozaki M., Nishiyama K., Saiki T., Watanabe S., and Tsuda Y. (2019) The geomorphology, color, and thermal properties of Ryugu: implications for parent-body processes. Science 364, doi:10.1126/science.aaw0422 PMC7370239 30890587

[B25] Tsuda Y., Yoshikawa M., Abe M., Minamino H., and Nakazawa S. (2013) System design of the Hayabusa 2—asteroid sample return mission to 1999 JU3. Acta Astronaut 91:356–362

[B26] Vernazza P., Marsset M., Beck P., Binzel R.P., Birlan M., Brunetto R., Demeo F.E., Djouadi Z., Dumas C., Merouane S., Mousis O., and Zanda B. (2015) Interplanetary dust particles as samples of icy asteroids. Astrophys J 806, doi:10.1088/0004-637X/806/2/204

[B27] Vinogradoff V., Bernard S., Le Guillou C., and Remusat L. (2018) Evolution of interstellar organic compounds under asteroidal hydrothermal conditions. Icarus 305:358–370

[B28] Watanabe S., Tsuda Y., Yoshikawa M., Tanaka S., Saiki T., and Nakazawa S. (2017) Hayabusa2 mission overview. Space Sci Rev 208:3–16

[B29] Yesiltas M. and Kebukawa Y. (2016) Associations of organic matter with minerals in Tagish Lake meteorite via high spatial resolution synchrotron-based FTIR microspectroscopy. Meteorit Planet Sci 51:584–595

